# Extracellular Vesicles Released From Cortical Neurons Influence Spontaneous Activity of Recipient Neurons

**DOI:** 10.1111/jnc.70231

**Published:** 2025-09-15

**Authors:** Franco Luis Lombino, Mohsin Shafiq, Andreu Matamoros‐Angles, Jürgen R. Schwarz, Kira V. Gromova, Daniele Stajano, Bente Siebels, Leonie Bergmann, Tim Magnus, Michaela Schweizer, Franz Lennard Ricklefs, Hartmut Schlüter, Andrew F. Hill, Matthias Kneussel, Markus Glatzel

**Affiliations:** ^1^ Institute for Neuropathology University Medical Center Hamburg‐Eppendorf Hamburg Germany; ^2^ Institute for Molecular Neurogenetics, ZMNH University Medical Center Hamburg‐Eppendorf Hamburg Germany; ^3^ Section Mass Spectrometry and Proteomics University Medical Center Hamburg‐Eppendorf Hamburg Germany; ^4^ Department of Neurosurgery University Medical Center Hamburg‐Eppendorf Hamburg Germany; ^5^ Department of Neurology University Medical Center Hamburg‐Eppendorf Hamburg Germany; ^6^ Core Facility of Morphology and Electron Microscopy, ZMNH University Medical Center Hamburg‐Eppendorf Hamburg Germany; ^7^ Institute for Health and Sport Victoria University Melbourne Australia

**Keywords:** cortex, extracellular vesicles, hippocampus, NMDA receptor, synaptic activity

## Abstract

Extracellular vesicles (EVs) are membranous structures that cells release into the extracellular space. EVs carry various molecules such as proteins, lipids, and nucleic acids, and serve as specialized transporters to influence other cells. In the central nervous system, EVs have been linked to many important processes, including intercellular communication, but molecular details of their physiological functions are not fully understood. Our study aimed to investigate how EVs are released by neuronal cells, and how they affect the neuronal activity of other recipient neurons. We show that mature primary cortical neurons release EVs from both their soma and dendrites. EVs released from neurons closely resemble non‐neuronal EVs regarding size and marker proteins, and proteomic analyses showed that neuronally released EVs contain proteins typically acting in pre‐ and post‐synaptic compartments. Interestingly, our analysis revealed that EVs alter spontaneous activity in target neurons by increasing the amplitude of postsynaptic potentials. In summary, our findings elaborate on the role of EVs in synaptic activity modulation in neurons mediated by glutamate receptors.

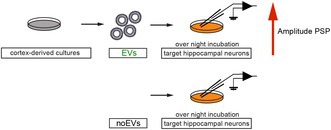

## Graphical abstract

Extracellular vesicles (EVs) are small, membrane‐bound particles released by cells that can transfer proteins, lipids, and nucleic acids to influence other cells. The roles of EVs in brain function are not well understood. We show that mature cortical neurons release EVs from both soma and dendrites, carrying proteins involved in synaptic signaling. These EVs enhance spontaneous activity in recipient neurons by increasing postsynaptic potential amplitude. They may have implications in inter‐neuronal communication, specifically in synaptic modulation in the brain.

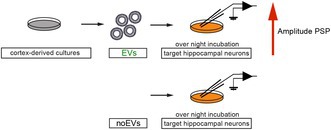

**Abbreviations**
AMPA: α‐amino‐3‐hydroxy‐5‐methyl‐4‐isoxazolepropionic acid
BCA: Bicinchoninic acid
BDEVs: brain‐derived extracellular vesicles
BDNF: brain‐derived neurotrophic factor
Cat. no.: catalog number
DIV: days in vitro
DNQX: 6,7‐dinitroquinoxaline‐2,3‐dione
E.16: embryonic day 16
EV: extracellular vesicles
F‐ACTIN: filamentous actin
FBS: fetal bovine serum
GFAP: glial fibrillary acidic protein
GFP: green fluorescent protein
HBSS: Hanks' balanced salt solution
lEVs: large extracellular vesicles
MAP2: microtubule‐associated protein 2
miRNA: micro ribonucleic acid
MVB: multivesicular body
NMDA
: *N*‐methyl‐D‐aspartic acid
NSE: neuronal specific enolase
NTA: nanoparticle tracking analysis
PBS: phosphate buffered saline
PCA: principal component analysis
PFA: paraformaldehyde
PSD95: post‐synaptic density protein 95
PSP: post‐synaptic potential
PVDF: polyvinylidene difluoride
sEVs: small extracellular vesicles
TBS: tris‐buffered saline
TEAB: triethylammonium bicarbonate buffer
TFA: trifluoroacetic acid
TIRF: total internal reflection fluorescence
UHPLC: ultra‐high performance liquid chromatography

## 1 Introduction

Extracellular vesicles (EVs) are released into the extracellular space by every cell type (Ibrahim and Khan 2025). They are a population of distinct membranous units and carriers of different types of biological material. Indeed, it is known that nucleic acids, proteins, and lipids can be transferred to cells using EVs as shuttles (Van Niel et al. 2018). EVs, mainly consisting of exosomes and microvesicles, are thought to have biologically relevant functions in cell‐to‐cell communication, and their therapeutic potential is of great interest (Claridge et al. 2021). Even if the molecular composition of specific EV subtypes is not fully uncovered, their molecular identity is thought to depend on the physiological state of the donor cells and the existence of specific tetraspanins in the EV membrane, which are also linked to specific functions of EVs (Karimi et al. 2022; Andreu and Yáñez‐Mó 2014).

In the brain, EVs are secreted by neurons, astrocytes, oligodendrocytes, and microglia (Brenna et al. 2020). Interestingly, EVs from different sources appear to have different functions in target cells (Budnik et al. 2016). In the central nervous system, EVs play key roles in brain development (Graykowski et al. 2020) and are directly involved in the spreading of pathogenic molecules in neurodegenerative diseases (Hill 2019).

Chemical synapses are central players in neuronal communication, and recent data indicate that EVs play roles in some aspects of synapse physiology. For instance, EVs derived from primary astrocytes impact neuronal synapse development (Patel and Weaver 2021) and the transfer of synaptic proteins such as synaptobrevin between neurons occurs via EVs (Vilcaes et al. 2021). Furthermore, AMPA (α‐amino‐3‐hydroxy‐5‐methyl‐4‐isoxazolepropionic acid) receptors are present in EVs, with neuronal activity regulating their secretion (Lachenal et al. 2011). Despite this evidence, the functional roles that neuronal EVs exert in neighboring recipient neurons are not fully uncovered. To better understand whether and how EVs secreted from donor neurons modulate synaptic activity in recipient neurons, we isolated and characterized cortical neuronal culture‐derived EVs, assessed their protein composition by mass spectrometry, and performed electrophysiological experiments in cultured hippocampal recipient neurons.

Here, we demonstrate that neurons release EVs not only from their soma but also from dendritic compartments. Moreover, we describe that the neuronal EVs enhance the amplitude of spontaneous post‐synaptic potentials in recipient neurons. Furthermore, our electrophysiological and proteomic analyses provide molecular cues on how EVs modulate synaptic activity, highlighting the role of glutamatergic signaling in this process.

## 2 Materials and Methods

### 2.1 Mice

Animals were housed under controlled environmental conditions according to all ethical regulations in agreement with the European Community Council Directive (2010/63/EU), reviewed and approved by the ethics committee of Hamburg (Behörde für Justiz und Verbraucherschutz, Fachbereich Lebensmittelsicherheit und Veterinärwesen—reference number N103/2024) and the animal care committee of the University Medical Center Hamburg‐Eppendorf (ORG844 and ORG1088). Animals of both sexes were used for experiments. They were housed in groups of 2 to 5 mice per cage and exposed to a 12‐h light–dark cycle. The animals were kept at a constant temperature (22°C ± 1°C) and humidity (50°C ± 5°C) and were provided with food and water ad libitum. Mice were sacrificed either with CO_2_ or decapitation for primary cultures.

### 2.2 Cortex‐Derived Neuronal Cell Cultures

C57Bl/6J (breeding colony at UKE, originally JAX cat. no. 000664) mouse embryos (5–8 embryos according to the litter size and the mother, for a total of 30 cultures considering replicates for all the experiments) were extracted from the uterine horns of pregnant female mice at E.16, and brains were harvested. Subsequently, cortices were isolated, pooled, and incubated for 5 min at 37°C in the presence of 0.005% Trypsin (Invitrogen, cat. no. 25300054) to promote dissociation. Later, trypsin was blocked with FBS (Invitrogen, cat. no. 10082139)/HBSS (Invitrogen, cat. no. 14170088), and all substituted by HBSS. Finally, further dissociation was achieved by a few passages through a glass pipette. For EV isolation, 0.7 cortices were plated on each 10 cm plate coated with poly‐L‐lysine (Sigma‐Aldrich, cat. no. P2636‐100 mg) the day before. In total, 6 to 10 plates were prepared for each isolation. For immunocytochemistry, cells were seeded on 12 mm glass coverslips (Carl Roth, P231.1) previously coated with poly‐L‐lysine.

### 2.3 Immunocytochemistry

Cortex‐derived primary cultures were fixed after DIV12 using 4% formaldehyde/PBS for 3–5 min. Later, cells were washed 3 times with PBS, permeabilized for 3–5 min with Triton X‐100/PBS, and washed further with PBS for three additional times. Subsequently, 1% BSA/PBS was used for blocking 30–60 min at room temperature. Primary antibodies were incubated in blocking solution for 1 h and washed 3 times. Finally, the secondary antibody was incubated in a blocking solution and, after washing thoroughly, coverslips were mounted with Aqua Poly‐Mount (Polysciences, cat. no. 18606‐20). Images were obtained with a spinning disk microscope (Visitron Systems GmbH) and processed using Fiji. Quantification was performed using the manual cell counter plugin of Fiji.

### 2.4 Time Lapse TIRF Microscopy

Cortical cultures were seeded on glass coverslips as above and maintained in culture until the young stage. Cells were transfected at DIV 6–7 with the Calcium Phosphate method using the CD63‐pHluorin construct from Addgene (cat. no. #130901) and mCherry as a volume marker. One day later, after replacing the media with fresh Hepes buffer, cells were imaged at the spinning disk microscope (Visitron Systems GmbH) in the presence of 5% CO_2_ and 37°C temperature. Images were acquired applying TIRF for 200 frames with an interval of 2 s, then analyzed on Fiji.

### 2.5 Extracellular Vesicle Isolation From Primary Cultures

EVs were isolated by a protocol developed by others (Laulagnier et al. 2017). In detail, cortex‐derived primary cultures were prepared as stated above and kept in culture for 12–14 days in vitro in 10 cm cell culture dishes, previously coated with poly‐L‐Lysine. On the experimental day, media were collected and kept on ice in the presence of protease inhibitors (Sigma‐Aldrich, cat. no. 04693132001). Cells were collected after scratching in ice‐cold Triton X‐100/PBS supplemented with protease inhibitors. Cell lysates were centrifuged for 10 min at 4°C at 1000 × *g*. Supernatant was conserved for further analysis. Media were centrifuged at 2000 × *g* 4°C for 10 min at 4°C. Later, the supernatant was collected and centrifuged at 20 000 × *g* for 30 min at 4°C. Cell debris was removed by filtration with a 200 nm filter and finally, flow through was centrifuged at 100 000 × *g* for 90 min at 4°C in SW40‐Ti swinging rotor. Supernatant was discarded. EVs were at the bottom of the tube and resuspended in filtered PBS containing protease inhibitors.

For *electrophysiological experiments*, the control condition was represented by primary hippocampal cultures administered with PBS containing protease inhibitors; in the tested condition, primary hippocampal cultures received EVs from primary cortical cultures resuspended in PBS plus protease inhibitors. All conditions received the same volume of material. Incubation was performed overnight in 300–400 μL of the primary hippocampal cultures pre‐conditioned media.

### 2.6 Isolation of Extracellular Vesicles From Mouse Brain

EVs were isolated from frozen brain tissues obtained from C57Bl/6J mice (JAX cat. no. 000664) (*n* = 4 animals; 35–50 weeks old) as previously described (Crescitelli et al. 2021). In brief, the tissue was mechanically minced and digested with DNAse I (40 U/mL, Roche, cat. no. 03724778103) and Collagenase D (2 mg/mL, Roche, LOT: 70507825) for 30 min at 37°C. To remove the cell and tissue debris, the samples were centrifuged at 300 × *g* and 2000 × *g*. Then, the supernatant was centrifuged at 16 000 × *g* to pellet the large EVs (lEVs), and later at 118 000 × *g* to pellet the small EVs (sEVs). The lEVs and the sEVs were mixed and centrifuged again in a three‐layer Optiprep cushion (Stemcell, cat. no. 07820) (45%, 30% and 10%) at 185 000 × *g*. The EVs were obtained from the fraction between the 30% and 10% layers. Western blots were performed as mentioned above.

### 2.7 Extracellular Vesicle Characterization Using Nanoparticle Tracking Analysis (NTA) and Western Blotting

*For NTA*: EV pellets obtained as previously described were diluted 1:1000 in sterile and filtered water for NTA measurements.

*For western blot*: EVs from different conditions were normalized based on Nanoquant measurements and BCA quantification, diluted with Laemmli buffer containing beta‐mercaptoethanol for all antigens, except for tetraspanins where urea‐containing Laemmli buffer was used. Finally, samples were either boiled or warmed up to 65°C, respectively, and loaded onto 10% gels and transferred to PVDF membranes via wet transfer using 20% methanol. Blocking was achieved in the presence of 3% Bovine Serum Albumin diluted in TBS‐0.1% Tween. All washing steps were performed in TBS‐0.1% Tween. Secondary HRP‐labeled antibodies were incubated for at least 1 h at room temperature (1:5000 in 3% BSA/TBS‐0.1% Tween) and imaged using Chemostar Touch v. 0.5.77 (INTAS Science Imaging Instruments GmbH) upon addition of the ECL substrate (BioRad cat. no. 1705061). Images were later visualized with Fiji.

### 2.8 Electron Microscopy and Immunogold Labeling

For pelleting, EVs from cortical cell cultures were ultracentrifuged at 100 000 × *g* for 70 min. The procedure was performed as in (Matamoros‐Angles et al. 2024). Shortly, the EVs were resuspended in 2% paraformaldehyde (PFA) for fixation. A fraction of this solution (5 μL) was adsorbed for 20 min to glow discharged carbon‐coated Formvar grids (EMS, Germany). After a brief washing step with PBS, samples were subjected to post‐fixation with 1% glutaraldehyde in PBS and incubated on ice‐cold methylcellulose‐uranyl acetate solution for 30 min. Grids were then looped out and air‐dried before analysis at the electron microscope.

For immunogold labeling, EVs were absorbed on grids as described above. Then, they were rinsed in PBS and quenched in 20 mM glycine in PBS. 1% bovine serum albumin (BSA) was used as a blocker for 10 min (Théry et al. 2006). Grids with EVs were incubated with the primary antibody GluA2 (AGC‐005, Alomone Labs) for 2 h. Protein A coupled to 10 nm colloidal gold particles was used to target the primary antibody (G. Posthuma, University Medical Center Utrecht) (1:50).

### 2.9 Patch‐Clamp Recordings From EVs‐Treated Hippocampal Neurons

Whole‐cell patch clamp measurements (Hamill et al. 1981) were performed on DIV12 to 14 hippocampal neurons. Neuron cultures were prepared from mouse embryos (E.16) as described above. Pipettes were made from borosilicate glass and had resistances of 3 to 5 MΩ after filling with intracellular solution (120 mM K‐gluconate, 8 mM NaCl, 2 mM MgCl_2_, 0.5 mM CaCl_2_, 5 mM EGTA, 10 mM HEPES, 14 mM phosphocreatine, 2 mM magnesium‐ATP, 0.3 mM sodium‐GTP, and pH adjusted to 7.3 with KOH). Patchmaster software (HEKA, Lambrecht, Germany) and an EPC‐9 patch‐clamp amplifier (HEKA) were used for data acquisition and pulse application. Recordings were low‐pass filtered at 2.9 kHz and analyzed with Fitmaster (HEKA, Lambrecht, Germany), Igor Pro 6.03 (Wavemetrics), and Excel (Microsoft). Neurons with an access resistance < 20 MΩ were evaluated. Current clamp recordings were performed on untreated cultured neurons and on neurons exposed to extracellular vesicles. Current clamp experiments were done at room temperature (21°C–23°C) in Ringer's solution (143 mM NaCl, 5 mM 1 KCl, 0.8 mM MgCl_2_, 1 mM CaCl_2_, 10 mM HEPES, 5 mM glucose, and pH adjusted to 7.3 with NaOH). All substances were purchased from Sigma Aldrich.

### 2.10 Mass Spectrometry‐Based Bottom‐Up Proteomics

#### 2.10.1 Tryptic Digestion

Samples were dissolved in 100 mM triethyl ammonium bicarbonate (TEAB) and 1% w/v sodium deoxycholate (SDC) buffer, boiled at 95°C for 5 min, and sonicated with a probe sonicator. The protein concentration of denatured proteins was determined by the Pierce bicinchoninic acid assay (BCA) Protein assay kit (Thermo Fisher) and samples were diluted to 10 μg of protein in 25 μL buffer. The samples were then pipetted into a 96‐well LoBind plate (Eppendorf, Hamburg, Germany) placed on an Andrew+ Pipetting Robot (Waters, Milford, USA), which was used to execute all following steps. Using the robot, disulfide bonds were first reduced in 10 mM dithiothreitol for 30 min at 56°C while shaking at 800 rpm and alkylated in the presence of 20 mM iodoacetamide for 30 min at 37°C, again while shaking at 800 rpm. Then, carboxylate modified magnetic E3 and E7 speed beads (Cytvia Sera‐Mag, Marlborough, USA) at a 1:1 ratio in LC–MS grade water were added in a 10:1 (beads/protein) ratio to each sample, following the single‐pot, solid‐phase enhanced sample preparation (SP3)‐protocol workflow 1. To bind the proteins to the beads, acetonitrile (ACN) concentration was raised to 50%. Subsequently, samples were shaken at 600 rpm for 18 min at room temperature. Magnetic beads were magnetized, and the supernatant was removed. Magnetic beads were further washed two times with 80% ethanol (EtOH) and then two times with 100% ACN. After resuspension in 100 mM AmBiCa, digestion with trypsin was performed (sequencing grade, Promega) at a 1:100 (enzyme: protein) ratio at 37°C overnight while shaking at 500 rpm. The next day, trifluoroacetic acid (TFA) was added to a final concentration of 1% to inactivate trypsin. The samples were then shaken at 500 rpm for 5 min at room temperature. Finally, beads were magnetized, and the supernatant containing tryptic peptides was transferred into a new 96‐well LoBind plate, ready for subsequent LC–MS/MS analysis.

#### 2.10.2 LC–MS/MS Measurement

Peptide separation was carried out using a two‐solvent system on a nano‐ultra‐high‐performance liquid chromatography (nano‐UHPLC) setup (Dionex Ultimate 3000 UHPLC, Thermo Fisher). Solvent A consisted of 0.1% formic acid in water, and solvent B was 0.1% formic acid in acetonitrile. The UHPLC was equipped with a C18 trap column (100 μm × 20 mm, 5 μm particle size, 100 Å pore size; Nano Viper, Thermo Fisher) for online desalting, followed by a 25 cm long C18 analytical column (75 μm × 250 mm, 1.7 μm particles, 130 Å pores; peptide BEH C18, nanoEase, Waters) for peptide separation. Chromatographic separation was achieved over an 80‐min method, ramping acetonitrile concentration from 2% to 30% over the first 60 min.

Mass spectrometric analysis was conducted using an Orbitrap Fusion quadrupole‐ion‐trap‐orbitrap instrument (Thermo Fisher), equipped with a nano‐electrospray ionization (nano‐ESI) source operating at 1800 V. Data were acquired in data‐independent acquisition (DIA) mode.

For each MS1 scan, ions were collected for up to 240 milliseconds or until an automatic gain control (AGC) target of 2 × 10^5^ ions was reached. High‐resolution MS1 spectra were recorded using the Orbitrap analyzer at 120 000 resolution (m/z 200), across a scan range of m/z 400–1200.

Within the DIA mode, MS2 fragmentations were conducted over the m/z 400–800 range using 12 m/z isolation windows with 1 m/z overlap. Collision‐induced dissociation (CID) was employed using a normalized collision energy of 30%. MS2 spectra were acquired at a resolution of 30 000 over a scan range of m/z 350–2000, using an AGC target of 5 × 10^4^ ions and a maximum injection time of 54 milliseconds.

#### 2.10.3 LC–MS/MS Data Processing and Analysis

LC–MS/MS data were searched with the CHIMERYS DIA algorithm integrated into the Proteome Discoverer software (v3.1.0.638, Thermo Fisher Scientific) against a reviewed murine Swissprot database using Inferys 3.0 fragmentation as the prediction model. Carbamidomethylation was set as a fixed modification for cysteine residues. The oxidation of methionine was allowed as a variable modification. A maximum number of one missing tryptic cleavage was set. Peptides between 7 and 30 amino acids were considered. A strict cutoff (FDR ≥ 0.01) was set for peptide identification. Quantification was performed by CHIMERYS based on fragment ions. The mass spectrometry proteomics data have been deposited to the ProteomeXchange Consortium via the PRIDE (doi: https://doi.org/10.1093/nar/gkae1011.) partner repository with the dataset identifier PXD062783.

For the statistical and bioinformatics analyses of the proteomics data sets, we relied on base R and various packages in R. Obtained protein abundances were log 2‐transformed and normalized by column‐median. Proteins which were detected in at least four replicates of a group (EV preparations and cell lysates individually) were kept for further analysis. Proteins were considered to be differentially regulated if the Student's *t*‐test‐based *p*‐value was ≤ 0.05 and at least a 1.5‐fold change in either direction was observed. Proteins were considered uniquely present in a certain group when they were expressed in at least four out of six replicates for that particular group and were simultaneously absent in the other group during pairwise comparison.

Venn diagram was prepared using *BioVenn* package (version 1.1.3, 2021). Correlation plots were prepared using *ggcorrplot* package (version 0.1.4, 2021). Pearson's coefficient values were obtained using base R. Initially, proteins with missing values were excluded from the Principal component analyses and heatmaps. Principal component analysis (PCA) was carried out using the *prcomp* function of the base R. Resultant PCA plot was composed utilizing the *ggplot2* package (version 3.3.3, 2016). Volcano plot was composed using *EnhancedVolcano* package (version 1.16.0, 2021) in R Studio.

Gene ontology (GO) analyses were performed utilizing the *clusterProfiler* (version 4.8.1, 2024), *enrichR* (version 3.2, 2024), org.Mm.eg.db (version 3.16.0, 2023), and *topGO* (version 2.50.0, 2023) packages. Overrepresentation analysis (ORA) was performed to find associations of differentially upregulated proteins with the GO category “Biological Process” with the following analysis parameters: *p*valueCutoff = 0.05, *q*valueCutoff = 0.2, *p*AdjustMethod = “BH”, minGSSize = 5, and maxGSSize = 500. Reactome pathway enrichment analyses were conducted using the *ReactomePA* package (version 1.14.0, 2021), using the ‘mouse’ database as a reference with the following analysis parameters: *p*valueCutoff = 0.05, *p*AdjustMethod = “BH”, *q*valueCutoff = 0.2, minGSSize = 10, and maxGSSize = 50. The protein interaction network was prepared using string‐db.org.

### 2.11 Statistical Analysis

A previous test to determine the sample size was not conducted, but the number of our experimental replicates mimics similar studies that obtained robust results (Patel and Weaver 2021). All the analyses were performed using GraphPad Prism version 10.0.2 (GraphPad Software Inc.). A normality test following D'Agostino and Pearson test was conducted throughout the study, with the exception of the analysis of Figure 2B. A normal distribution allowed us to perform an unpaired *t*‐test, and a *p*‐value lower than 0.05 was considered significant. Also, *t* and df values are reported in the figure legends. For the LC–MS/MS data analysis, see the dedicated paragraph in the methods section.

## 3 Results

### 3.1 Cortex‐Derived Primary Cultures Display Active Synapses and Release Extracellular Vesicles

**FIGURE 1:**
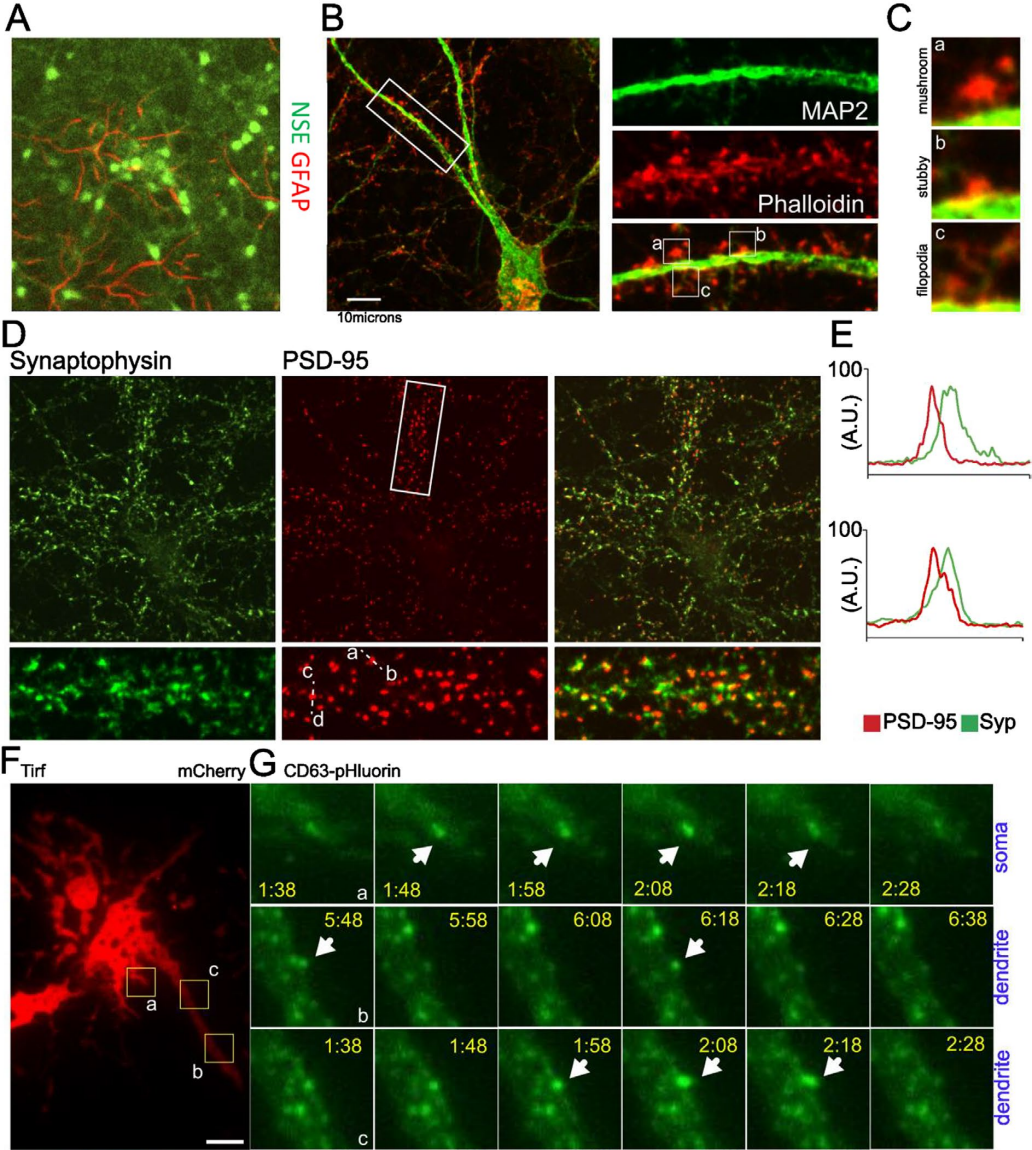
Cultured cortical neurons secrete extracellular vesicles. (A) Immunofluorescence staining of primary neurons derived from mouse cortex. NSE (green) and GFAP (red) coexistence indicates the presence of both glial cells and primary neurons (*n* = 2 independent cultures). (B) Primary neurons co‐stained with MAP2 and Phalloidin to observe postsynaptic spines in neuronal dendrites. Scale bar 10 μm (*n* = 3 independent cultures). (C) MAP2 and Phalloidin staining shows the presence of mushroom (a), stubby (b) and filopodia (c) spines in B. (D) Co‐staining of the presynaptic marker synaptophysin (Syp) and the postsynaptic marker PSD95 indicates the presence of active glutamatergic synapses. (*n* = 3 independent cultures). (E) Overlapping profile of the co‐staining synaptophysin and PSD95 shown in D. A.U. is arbitrary units. (F) Single frame of timelapse microscopy showing the overexpression of the volume marker mCherry for Tirf microscopy experiment Scale bar 10 μm. (*n* = 3 independent cultures). (G) Magnification of regions indicated in F. he arrows indicate a brief exosomal release event positive for CD63‐pHluorin. Time stamp minutes: seconds in yellow.

To evaluate the role of EVs in synaptic modulation, we used cortex‐derived primary neuronal cultures from mouse embryos. To verify the cellular identity in our primary culture model, we stained DIV14‐15 cultures with antibodies against both neuronal‐specific enolase (NSE) as a neuronal marker and glial fibrillary protein (GFAP) as a marker for astrocytes. Immunostaining confirmed that our model represents a mixed culture consisting of 72.37% ± 3.03% of primary neurons and 27.63% ± 3.03% of astrocytes (Figure 1A and Figure S1A,B). Next, we tested if neurons displayed structures typically observed in mature neurons and co‐stained the microtubule‐associated protein 2 (MAP2) and the F‐actin filaments with rhodamine‐phalloidin to detect dendrites and dendritic spines. Indeed, our neurons contain synaptic compartments (Figure 1B) that can be clustered into filopodia, stubby, and mushroom spines (Figure 1C). To test if cortical neurons at DIV14 were able to form mature synapses, we co‐stained against synaptophysin (pre‐synaptic marker) and the post‐synaptic density protein 95 (PSD‐95, post‐synaptic marker) (Figure 1D). The co‐localization of both markers indicated the presence of mature excitatory glutamatergic synapses (Figure 1E). In summary, our cell culture model has features of mature cortical neurons in coexistence with glial cells, being a suitable in vitro model to study the role of EVs in neuronal activity. To assess if primary cortical neurons spontaneously release EVs, we utilized a previously characterized CD63‐pHluorin construct to monitor the fusion of the multi vesicular bodies (MVB) to the plasma membrane and GFP fluorescence as an indication of EV release (Verweij et al. 2018). In combination with a volume marker to observe cellular morphology (Figure 1F and Figure S1C), we performed TIRF microscopy. We distinguished two types of GFP clusters (Figure 1F,G): membrane‐stable clusters and dynamic clusters representing release events (Figure 1G arrowheads). As expected from a protein of the tetraspanin family, a significant fraction of CD63 remains immobile in the cell membrane, likely in tetraspanin‐enriched microdomains. However, MVB fusion and, therefore, EV release occurred both in the cell soma and in dendritic structures (Figure 1F,G and videos in Data S6).

**FIGURE 2:**
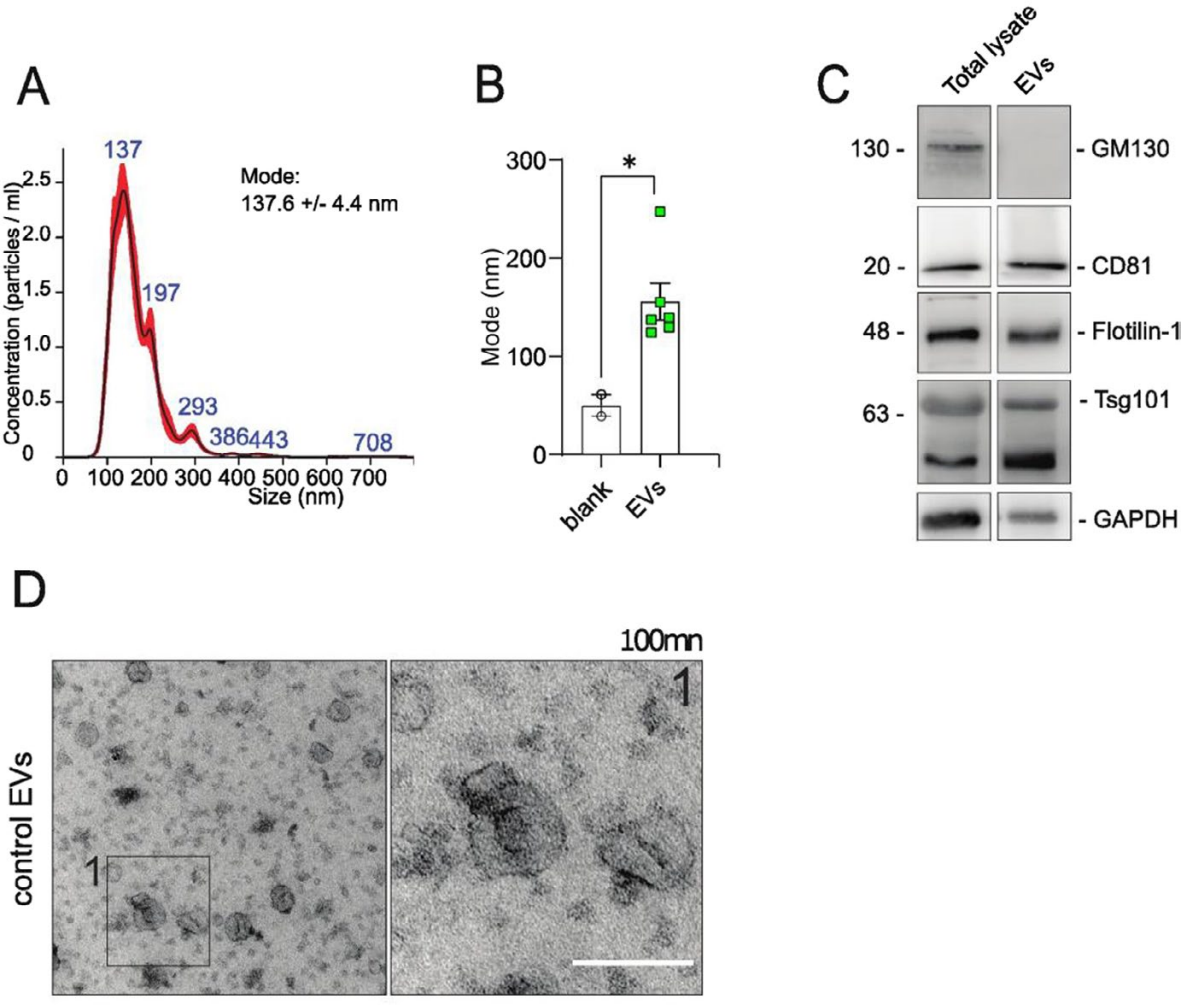
EV isolation from cultured cortical neurons. (A) Nanoparticle tracking analysis representative example of extracellular vesicles isolated via differential centrifugation. (B) Average mode size of EVs isolated from primary cultures reflects the expected particle size as compared to the control buffer. Blank *n* = 2 blanks, average 50.10 ± 10.90 SEM; EVs *n* = 6 preparations, average 155.6 ± 18.87 SEM; no normal distribution assessed; Unpaired *T*‐test; *p* = 0.0231*; *t* = 3.029, DF = 6; no values excluded, no test for outliers conducted. (C) Characterization of isolated EVs by western blot shows the presence of markers typically present in EVs (CD81, Flotillin‐1 and Tsg101) and the lack of GM130 (negative control); *n* = 3 independent preparations. (D) Electron microscopy of purified EVs showing the presence of particles of around 100 nm in diameter as well as smaller vesicles. Scale bar, 100 nm. Error bars: SEM.

From the same neuronal cultures, EVs were isolated and characterized following the MISEV2023 guidelines (Welsh et al. 2024). EVs were purified via differential centrifugation, and Nanoparticle Tracking Analysis (NTA) measurements showed an EV population with an average size of 155.6 ± 18.87 nm (Figure 2A,B). Further characterization of EVs by western blotting with antibodies to GM130 (a Golgi apparatus marker, used as an EV purity marker) and EV markers CD81, Tsg101, and Flotillin‐1 validated the purity of our EV isolation (Figure 2C and Figure S2). Lastly, we performed transmission electron microscopy to further validate EVs' morphology and shape, which showed the characteristic membranous EV cup shape (Figure 2D). In summary, our EV preparations from mature cortex‐derived primary neuronal cultures were highly enriched for intact EVs of 70–150 nm size in diameter that expressed membranous and intraluminal EV markers. This prompted us to further investigate the makeup and function of these EVs.

### 3.2 The Proteomic Analysis of Cortical Culture‐Derived EVs Reveals That They Are Carriers of Neuromodulatory Proteins

**FIGURE 3:**
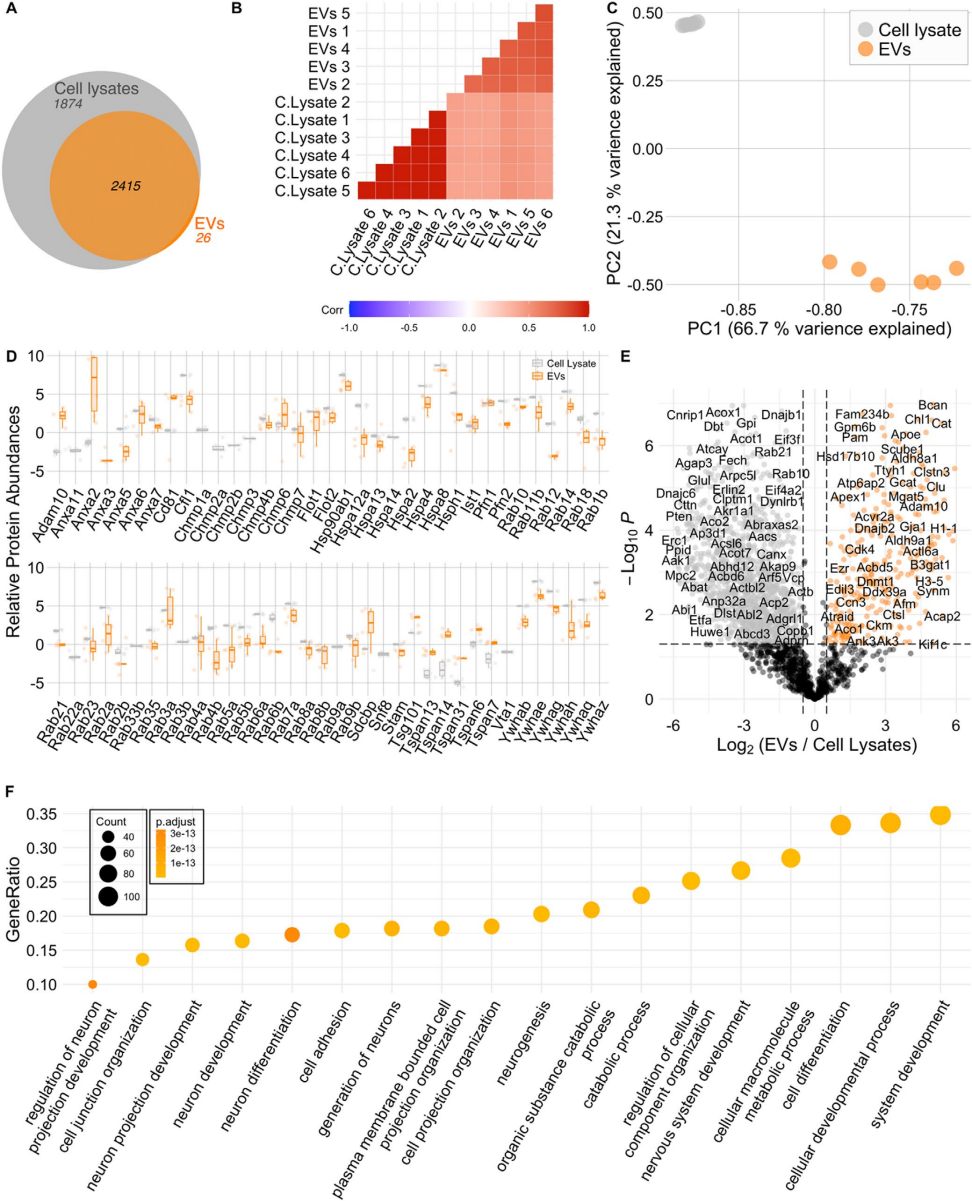
Proteomic composition of EVs derived from Primary neurons. (A) Venn diagram depicts a major overlap in proteomic composition of EVs sourced from primary neurons to that of respective cellular lysates. List of the proteins uniquely identified in the proteomics data set of EVs is included as Table S1. The correlation plot (B) (with high Pearson's correlation values) and Principle Component Analysis—PCA (C) underscore the internal consistency within the EV samples and lysates, highlighting the degree of similarity in their protein expression patterns. Calculation of correlation values and PCA were conducted for the proteins which were commonly expressed between the EVs and lysates. (D) Relative abundances of various EV marker proteins in the EVs and Lysates are depicted graphically. The abundance of these markers confirms the purity of the isolated EV population, crucial for accurate interpretation of subsequent analyses. (E) Volcano plot showing upregulated and downregulated proteins in EVs compared to Lysates depicting the proteins preferentially packed in the EVs (highlighted orange) with respect to the donor lysates. (F) The dotplot showcases the top gene ontology terms (category: Biological processes), that are linked to the proteins predominantly found within EVs.

**FIGURE 4:**
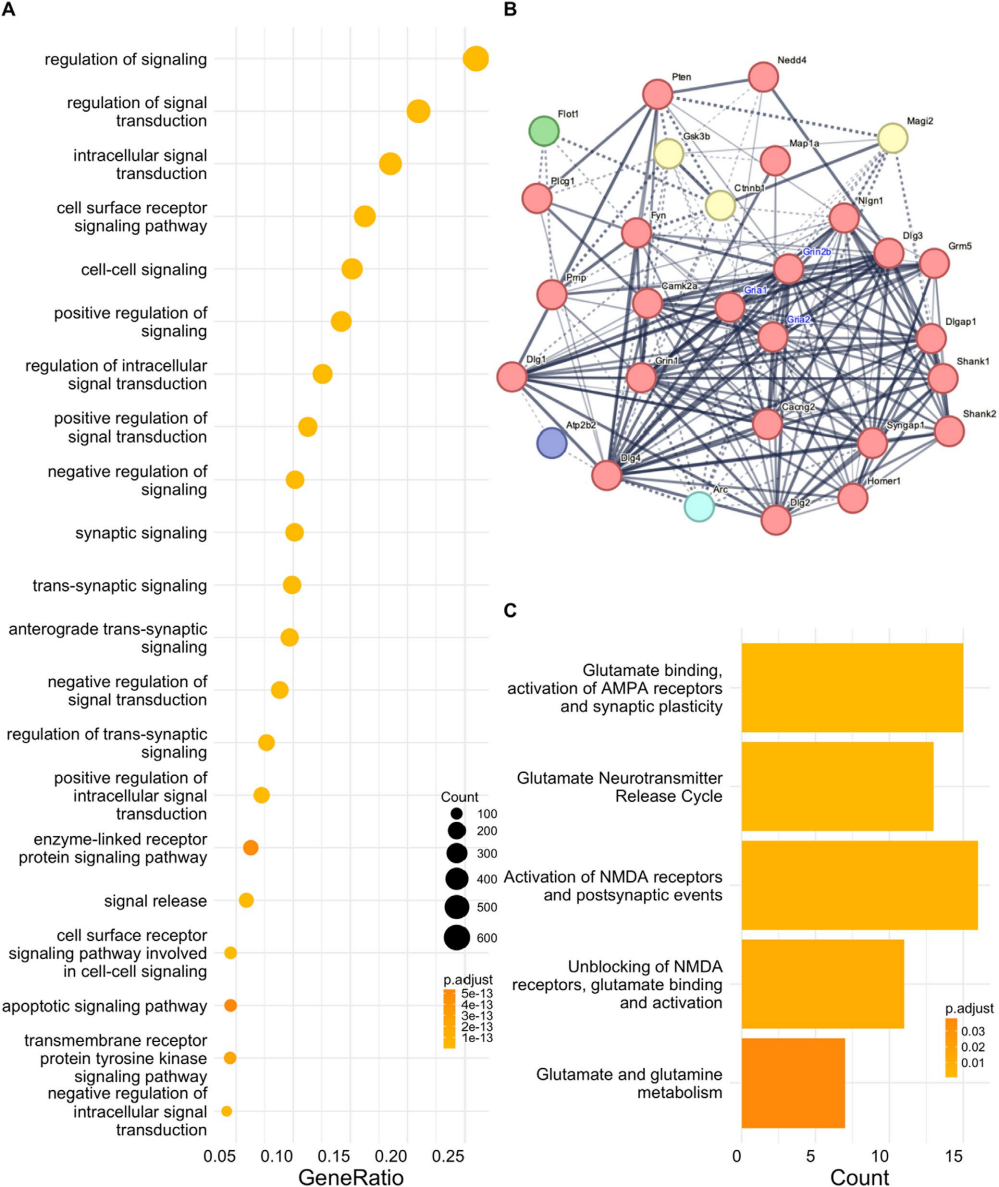
Certain proteins in primary culture derived EVs were associated to cell–cell signaling, trans‐synaptic signaling, and NMDA receptor trafficking and activity. (A) The dot plot presented here illustrates diverse signaling (Synaptic and receptor protein kinase) pathways identified through the GO enrichment analysis. List of the proteins is presented in Data S3. (B) Network of the EV proteins (from the proteomics data) related to Glutamate receptor binding pathway is shown here. Network was assorted together with String‐db application in cytoscape. (C) Reactome pathway enrichement for the proteins expressed in the EVs shows their active involvement in the AMPA receptor and NMDA receptor trafficking depicted here by dotplot (Data S4).

**FIGURE 5:**
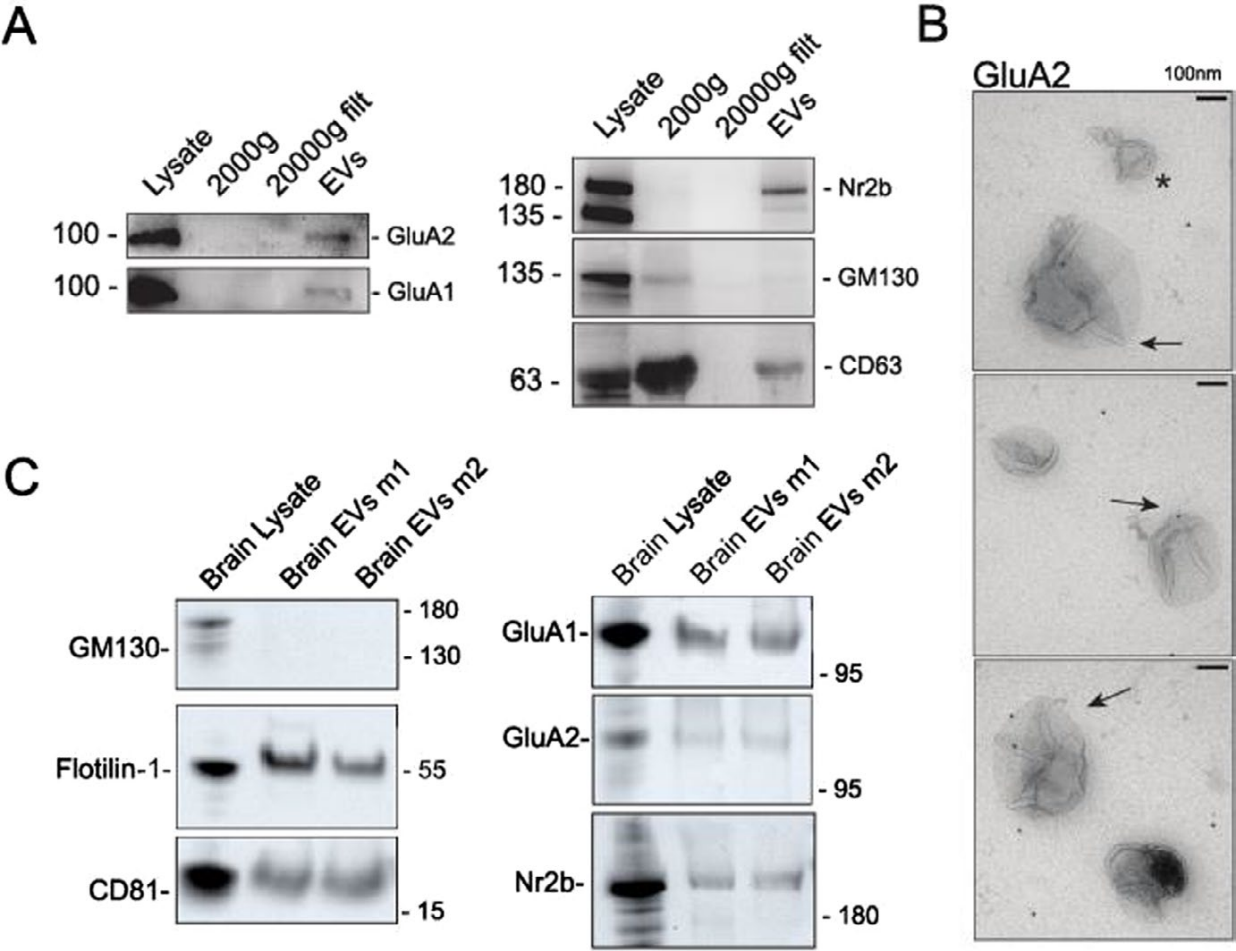
EVs isolated from primary cultures contain AMPA receptor subunits. (A) Glutamate receptor subunits are present in EVs isolated from primary cultures, in combination with the EV‐marker CD63. (B) Immunoelectron microscopy of EVs isolated from cultured neurons support the presence of the glutamate receptor subunit GluA2. (C) EVs isolated from mouse brains (*n* = 4 mice) and validated qualitatively by the presence of the EV markers Flotillin‐1 and CD81, contain both AMPA receptor subunits and Nr2b subunit of NMDA receptors. SIgnificance use of * indicates GluA2‐negative EV.

To assess whether neuronal EVs are enriched in proteins involved in synaptic function, we performed global proteomics of EVs and corresponding cell lysates (*n* = 6 in both) (Figure 3). Except for 26 proteins found exclusively in EVs, we found 2415 proteins overlapping between EVs and lysates, as shown in the Venn diagram that depicts all the detected proteins across samples (Figure 3A). We also found a high degree of intragroup similarity between the lysate samples compared with the EVs, and vice versa, showing a higher correlation with the samples from the same origin (Figure 3B,C). Moreover, the presence of several known EV markers shown in Figure 3D argues in favor of the enrichment and purity of our EVs preparations. The volcano plot (Figure 3E) comparing the protein abundances in EVs preparations and lysates shows a variety of proteins significantly enriched in EVs, such as Adam10, Apoe, and Ezr; (in orange) and those preferentially present in the donor cells' lysates (in gray). Gene ontology (GO) analysis highlighting the biological processes in which proteins preferentially found in EVs are involved reveals roles primarily related to neuronal development, proliferation, and differentiation (Figure 3F, Data S2). Furthermore, we looked for the potential involvement of EV proteins in synaptic and intracellular signaling processes (Figure 4) by performing GO (Data S3) and Reactome pathway enrichment analyses (Data S4). The GO enrichment analyses (Figure 4A) argue in favor of EVs being involved in synaptic and trans‐synaptic signaling, primarily driven by the presence of glutamate receptors (evident from the presence of glutamate receptors subunits GluA1, GluA2 and GluN2b in the data) and their interactors CamkIIα and Gsk3β (Figure 4B,C). Interestingly, the presence of AMPA receptors and NMDA receptors in our EVs could be confirmed by western blot of EV fractions derived from neuronal cultures (Figure 5A). Moreover, using immunoelectron microscopy, AMPA receptor GluA2 could be mapped to the outer leaflet of the EV lipid bilayer (Figure 5B). To assess the in vivo relevance of our data, we evaluated the presence of AMPA and NMDA receptors in brain‐derived EVs (BDEVs) obtained from mouse brain (Crescitelli et al. 2021). The BDEVs were of high purity as shown by NTA and TEM (Figure S3). These analyses confirmed the presence of AMPA receptor subunits (GluA1, GluA2) and NMDA receptor subunit Nr2b on EVs (Figure 5C).

### 3.3 EVs Derived From Cortical Cultures Modulate the Synaptic Activity of Recipient Hippocampal Cultures

**FIGURE 6:**
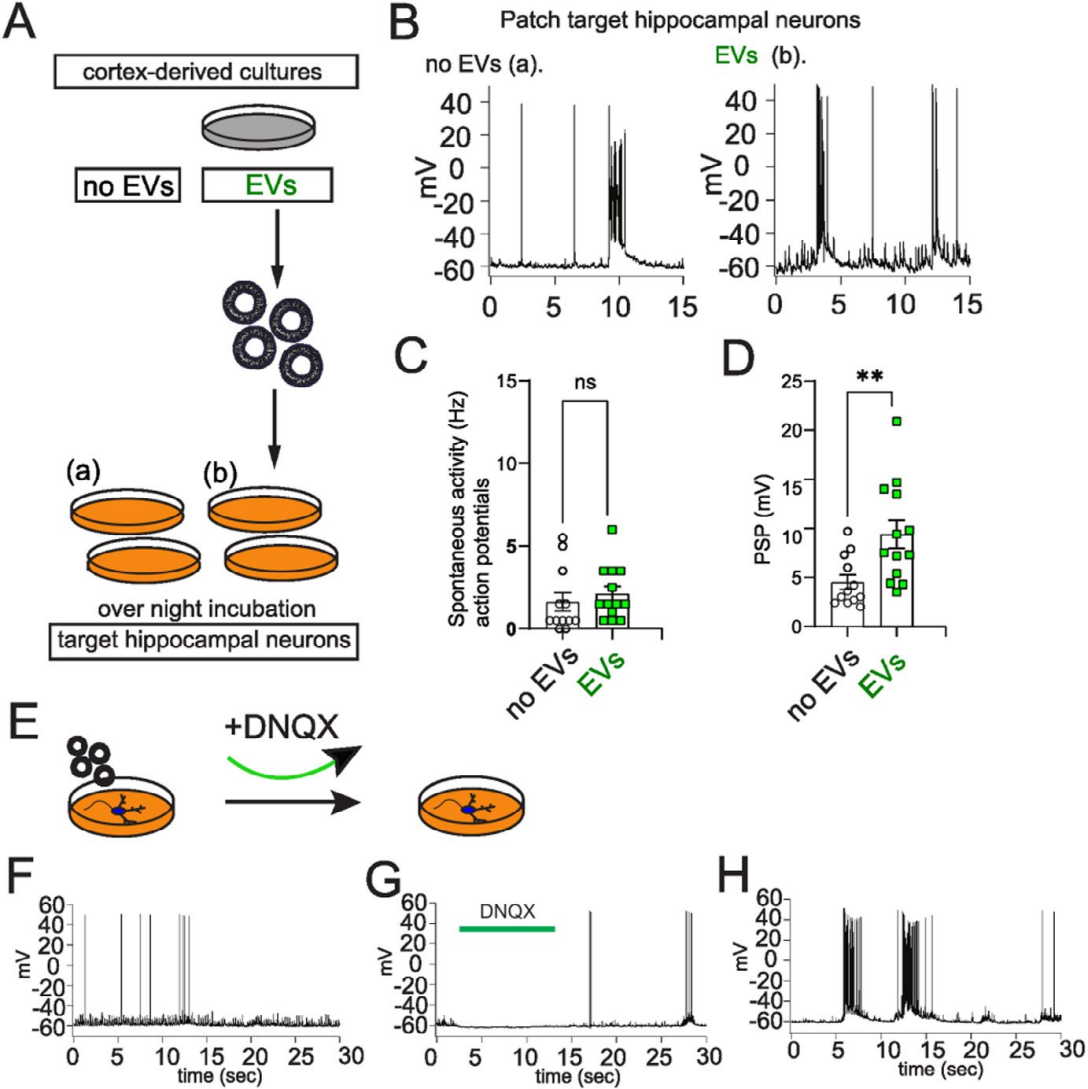
Extracellular vesicles modulate the synaptic activity of target cells. (A) Strategy and conditions of EVs administration protocol for electrophysiological recordings. Briefly, EVs from cultured cortical neurons were administered to hippocampal primary cultures overnight. Patch clamp recordings were performed the day after. (B) Example traces of patch clamp recordings of hippocampal cultures. Left panel “a” shows a recording from a neuron of hippocampal cultures which received only vehicle solution (PBS/inhibitors). Panel “b” in the right shows a recording of a neuron of hippocampal cultures which received EVs from non‐stimulated cortical cultures. (C) Quantification of frequency of action potentials show no significant difference between conditions. (*n* = 3 independent donor cortical and acceptor hippocampal cultures). (D) Amplitude of events is significantly affected by the presence of EVs. No EVs *n* = 12 recordings, 3 independent donor cortical and acceptor hippocampal cultures; average 4.517 ± 0.7406 SEM; EVs *n* = 13 recordings, 3 independent donor cortical and acceptor hippocampal cultures; average 9.377 ± 1.417 SEM; normal distribution D'Agostino and Pearson test, unpaired *T*‐test *p* = 0.0069**, *t* = 2.965, DF = 23; no values excluded, no test for outliers conducted. (E–H) Shows that application of DNQX abolishes postsynaptic potentials and its subsequent washing leads to a re‐activation of synaptic activity, implicating that primarily excitatory currents were recorded. Error bars: SEM.

Aiming to understand the role of neuronally released EVs in synaptic activity, we applied EVs derived from primary cortical cultures to DIV14 hippocampal neuron cultures. After overnight incubation (Figure 6A), we performed current‐clamp recording and evaluated the frequency of spontaneously firing action potentials and the amplitude of postsynaptic potentials. As shown in Figure 6B–D, EVs did not alter the frequency of synaptic potentials when compared to the no‐EV control (Figure 6B,C), but led to significantly increased amplitudes in post‐synaptic potentials (PSP) (Figure 6B,D). Additionally, the application of DNQX (a potent and selective AMPA/kainate‐receptor antagonist) to the recipient neurons markedly reduced neuronal activity, and the subsequent washing out of the DNQX led to a reactivation of the synaptic activity (Figure 6E,F). Together, these results indicate that EVs are synaptic modulators and influence post‐synaptic potentials in recipient cells.

In summary, our data strongly argue in favor of a prominent contribution of EVs in modulating synaptic activity in recipient cells, and based on our mass spectrometry data, we propose the involvement of glutamate receptors in this process.

## 4 Discussion

EVs have been related to numerous cellular processes in the central nervous system (Gassama and Favereaux 2021). Likely because of the diversity of cells that secrete functionally different subtypes of EVs (Lizarraga‐Valderrama and Sheridan 2021), their impact on target cells can be highly heterogeneous. For instance, lipids transported in EVs are known to alter target cell membrane characteristics (Record et al. 2018). Moreover, proteins contained in EVs can regulate EV biogenesis, secretion, or uptake by target cells (Lizarraga‐Valderrama and Sheridan 2021; Stajano et al. 2023). Here we show that secretion of EVs occurs at multiple sites within neurons, including dendrites and soma. Although in dividing cells, this may have minor implications, in large and highly polarized neurons, characterized by a soma, a complex dendritic tree, and an axon, each one with highly diverse molecular and electrical properties, secretion from different compartments may affect the molecular makeup and function of the secreted EVs (Blanchette and Rodal 2020; Huo et al. 2021). In this respect, it was shown that MVBs, the source of the exosomes, are widely distributed in both soma and dendrites of neurons in vivo and in vitro (Von Bartheld and Altick 2011).

In this study, we revealed that EVs impact the amplitude of post‐synaptic potentials (PSP) in target cells, and the blockage of neuronal activity via DNQX suggests that the major component transferred by cortical EVs was excitatory, from the glutamatergic system. Similarly, it was recently shown that vesicles released by glioma cells impact synchrony in neurons (Spelat et al. 2022). In this study, the authors showed that in addition to increasing Arp3 protein levels, exosomes derived from glioma cells increased the number of excitatory synapses in recipient neurons. In another study, the authors characterized the roles of exosomes and the larger microvesicles in modulating the spontaneous activity of cortical neurons (Brás et al. 2022), and although a complete understanding of the different roles of these two populations in target cells was not achieved, the study adds additional evidence to the role of EVs in modulating brain function. How do EVs lead to an increase in PSP? Uptake of BDNF and TrkB receptor‐containing EVs by clathrin‐dependent endocytosis by neurons has been shown to modulate synapse density through TrkB receptor activation (Solana‐Balaguer et al. 2023). Thus, it is conceivable that the equilibrium of glutamate receptors responsible for fine‐tuning post‐synaptic potentials in neurons is altered by the uptake and membrane integration of glutamate receptors contained in EVs (Kwok et al. 2021). Along the same line, recent work showed that neuron‐derived EVs increase excitatory synapse formation also in hippocampal neurons due to the delivery of certain miRNAs by the EVs, which could also explain our results (Antoniou et al. 2023).

Aiming to uncover molecular contributors mediating synaptic changes in target hippocampal neurons, we performed mass‐spectrometry analysis of EVs and lysates from donor cortical neurons. Interestingly, we identified a variety of proteins involved in neuronal development and pre‐ and post‐synaptic regulation in EVs. These include proteins responsible for modulating post‐synaptic potentials. In parallel, our results show that EVs from cortical neurons contain AMPA and NMDA receptors. Others have shown that AMPA receptors are frequent cargoes of EVs (Fauré et al. 2006; Olivero et al. 2021), and we confirmed that both are present via western blot and immunoelectron microscopy. In parallel, we supported our results by showing the presence of both AMPA and NMDA receptor subunits in EVs obtained from mouse brain tissue. In recent work, the proteomic analysis of brain‐derived EVs from mouse and human tissue revealed a prominent expression of proteins involved in postsynaptic density and specialization and similar synaptic functions (Matamoros‐Angles et al. 2024). Data on the presence of NMDA receptor subunits in EVs is scarce. One study by Fauré and others (Fauré et al. 2006) observed the presence of AMPA receptor subunits, but not the GluN1 subunit of NMDA receptors in cortical cultures derived EVs. Others have found the GluN1 subunit and other glutamate receptors in EVs in exosomes derived from human plasma (Looze et al. 2009; Goetzl et al. 2018). GluN2b is likely in distinct vesicles that are a part of those containing AMPA receptor subunits. Since NMDA receptors are implicated in neuronal maturation, our findings highlight the putative role of EVs in modulating neuronal development and maturation (Hou and Zhang 2017). Our understanding of the membrane topology of neuronal receptors in EVs is limited. However, it is reasonable to assume that, since the membrane topology of EVs reflects that of their parent neurons, these receptors are likely presented on the outer leaflet of the EV lipid bilayer.

While our study is subject to limitations inherent to in vitro models, including the potential confounding effects of astrocyte‐derived EVs (Zhao et al. 2021), it advances our understanding of EVs as modulators of neural communication and brain function. Specifically, our findings demonstrate that EVs modulate specific components of synaptic activity and contribute to the maintenance of target neuron synaptic properties. Furthermore, we provide an initial observation into the potential role of EVs in the cross‐regional regulation of synaptic activity, suggesting that EVs derived from cortical primary neurons can influence synaptic function in hippocampal primary neurons, although further validation is needed.

## 5 Conclusion

In summary, we show that mature primary cortical neurons release EVs from both soma and dendrites, which resemble non‐neuronal EVs in size and marker composition and contain synaptic proteins. These EVs enhance spontaneous activity in target neurons by increasing the amplitude of postsynaptic potentials.

## Author Contributions

**Franco Luis Lombino:** conceptualization, investigation, writing – original draft, formal analysis, writing – review and editing, methodology, data curation. **Mohsin Shafiq:** conceptualization, investigation, writing – original draft, writing – review and editing, formal analysis, methodology, data curation. **Andreu Matamoros‐Angles:** investigation, writing – original draft, writing – review and editing, formal analysis, methodology, data curation. **Jürgen R. Schwarz:** investigation, writing – review and editing, data curation. **Kira V. Gromova:** investigation, writing – review and editing. **Daniele Stajano:** investigation, writing – review and editing. **Bente Siebels:** investigation, formal analysis, methodology, data curation. **Leonie Bergmann:** investigation. **Tim Magnus:** investigation, writing – review and editing. **Michaela Schweizer:** investigation. **Franz Lennard Ricklefs:** investigation, conceptualization, formal analysis. **Hartmut Schlüter:** investigation, methodology. **Andrew F. Hill:** conceptualization, writing – review and editing, investigation. **Matthias Kneussel:** conceptualization, writing – original draft, writing – review and editing, funding acquisition, formal analysis, supervision, methodology. **Markus Glatzel:** conceptualization, funding acquisition, writing – original draft, writing – review and editing, supervision, formal analysis, methodology.

## Conflicts of Interest

The authors declare no conflicts of interest.

## Peer Review

The peer review history for this article is available at https://www.webofscience.com/api/gateway/wos/peer‐review/10.1111/jnc.70231.

## Supporting information


**Data S1:** jnc70231‐sup‐0001‐DataS1.xlsx.

**Data S2:** jnc70231‐sup‐0002‐DataS2.xlsx.

**Data S3:** jnc70231‐sup‐0003‐DataS3.xlsx.

**Data S4:** jnc70231‐sup‐0004‐DataS4.xlsx.

**Figure S1:** Primary cultures from the mouse cortex contain both neurons and astrocytes. (A) Immunocytochemistry against Neuronal Specific Enolase (NSE) and GFAP (astrocytes) depicts the presence of both cell types. (B) Manual quantification of the percentage of NSE+ and GFAP+ cells as an indication of neurons and astrocytes respectively. NSE+ cells *n* = 11 fields of view, average percentage 72.37 ± 3.033 SEM; GFAP+ cells *n* = 11 fields of view average percentage 27.63 ± 3.033 SEM; normal distribution D'Agostino and Pearson test, Unpaired *t*‐Test ****p ≤ 0.0001; *t* = 10.43, DF = 20; no values excluded, no test for outliers conducted. (C) Single frame of the Tirf microscopy experiment showing CD93‐pHluorin expression and relative to Figure 1F. Error bars: SEM.
**Figure S2:** Related to Figure 2C. Western blot showing the lanes selected for visualization in Figure 2C.
**Figure S3:** The BDEVs show the expected size distribution profile and morphology: (A) Representative NTA of the BDEVs shown in Figure 4. The BDEVs showed the usual normal‐like size distribution with an average diameter of 155 nm. (B) Representative TEM image of the BDEVs showing the membrane of the vesicles and their characteristic cup‐shape. Scale bar = 200 nm.
**Table S1:** List of 26 EVs proteins detected uniquely in the proteome of EVs.

**Data S6:** jnc70231‐sup‐0006‐DataS6.zip.

## Data Availability

The authors confirm that the data that supports the findings hereby presented are included within this manuscript. Also, the proteomics data was deposited to the ProteomeXchange Consortium via the PRIDE (doi: 10.1093/nar/gkae1011.) partner repository with the dataset identifier PXD062783.
