# Supplementary material for: Extracellular Vesicles Released From Cortical Neurons Influence Spontaneous Activity of Recipient Neurons
Source: J Neurochem. 2025 Sep 15;169(9):e70231. doi: 10.1111/jnc.70231 (PMC12435116; doi:10.1111/jnc.70231)
Supplement: Supplementary file 5 — Figure S1: Primary cultures from the mouse cortex contain both neurons and astrocytes. (A) Immunocytochemistry against Neuronal Specific Enolase (NSE) and GFAP (astrocytes) depicts the presence of both cell types. (B) Manual quantification of the percentage of NSE+ and GFAP+ cells as an indication of neurons and astrocytes respectively. NSE+ cells n = 11 fields of view, average percentage 72.37 ± 3.033 SEM; GFAP+ cells n = 11 fields of view average percentage 27.63 ± 3.033 SEM; normal distribution D'Agostino and Pearson test, Unpaired t‐Test ****p ≤ 0.0001; t = 10.43, DF = 20; no values excluded, no test for outliers conducted. (C) Single frame of the Tirf microscopy experiment showing CD93‐pHluorin expression and relative to Figure 1F. Error bars: SEM. Figure S2: Related to Figure 2C. Western blot showing the lanes selected for visualization in Figure 2C. Figure S3: The BDEVs show the expected size distribution profile and morphology: (A) Representative NTA of the BDEVs shown in Figure 4. The BDEVs showed the usual normal‐like size distribution with an average diameter of 155 nm. (B) Representative TEM image of the BDEVs showing the membrane of the vesicles and their characteristic cup‐shape. Scale bar = 200 nm. Table S1: List of 26 EVs proteins detected uniquely in the proteome of EVs. [file JNC-169-0-s004.pdf]

Figure 1

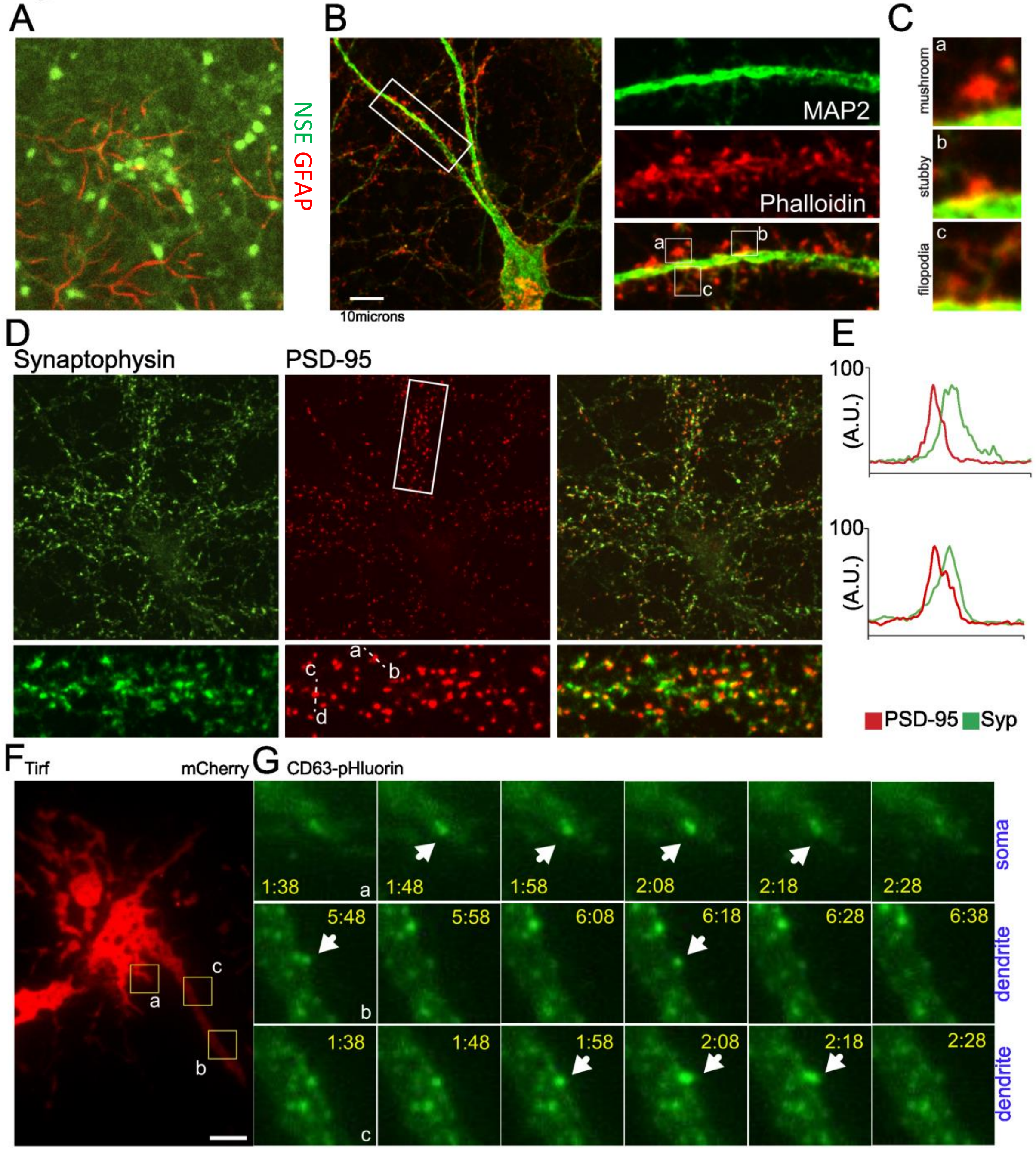
A
NSE GFAP
B
10microns
MAP2
Phalloidin
a
b
c
C
mushroom
stubby
filopodia
D
Synaptophysin
PSD-95
a
b
c
d
E
100
(A.U.)
100
(A.U.)
PSD-95 Syp
F Tirf
mCherry
G CD63-pHluorin
1:38 1:48 1:58 2:08 2:18 2:28
5:48 5:58 6:08 6:18 6:28 6:38
1:38 1:48 1:58 2:08 2:18 2:28
soma
dendrite
dendrite

506

Figure 2

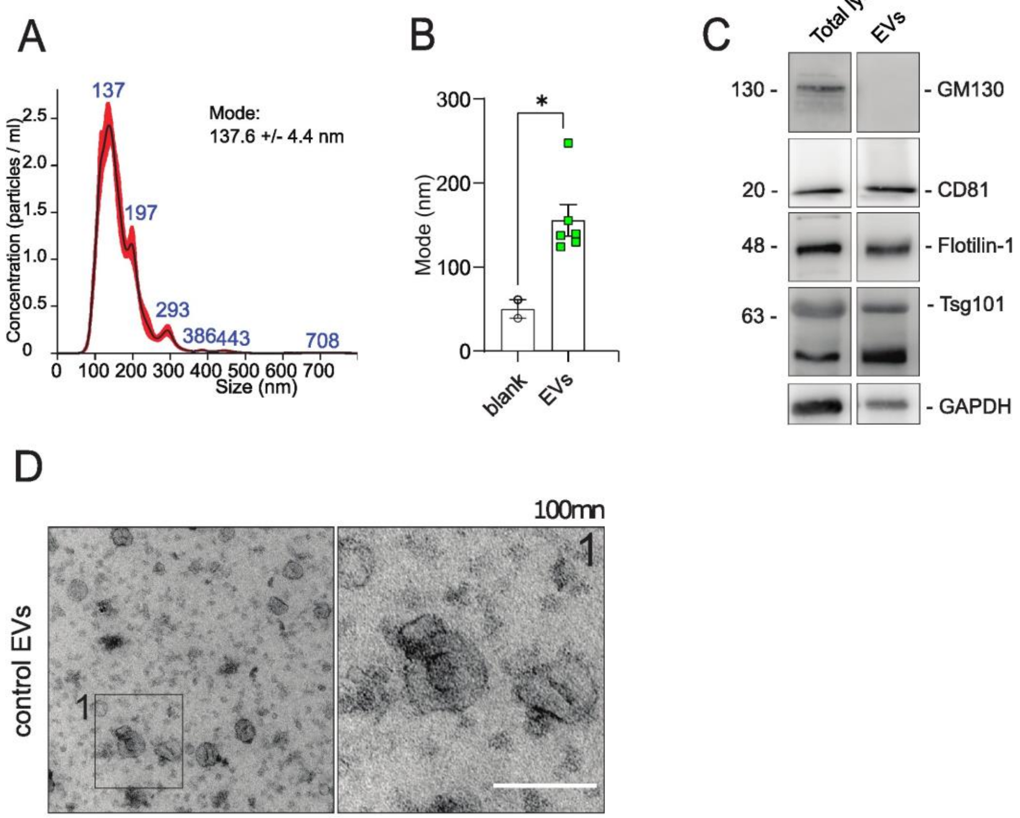
A
137
Mode:
137.6 +/- 4.4 nm
197
293
386
443
708
Concentration (particles / ml)
Size (nm)
B
*
Mode (nm)
blank
EVs
C
Total lysate
EVs
130 -
- GM130
20 -
- CD81
48 -
- Flotilin-1
63 -
- Tsg101
- GAPDH
D
100mn
1
control EVs

Figure 3

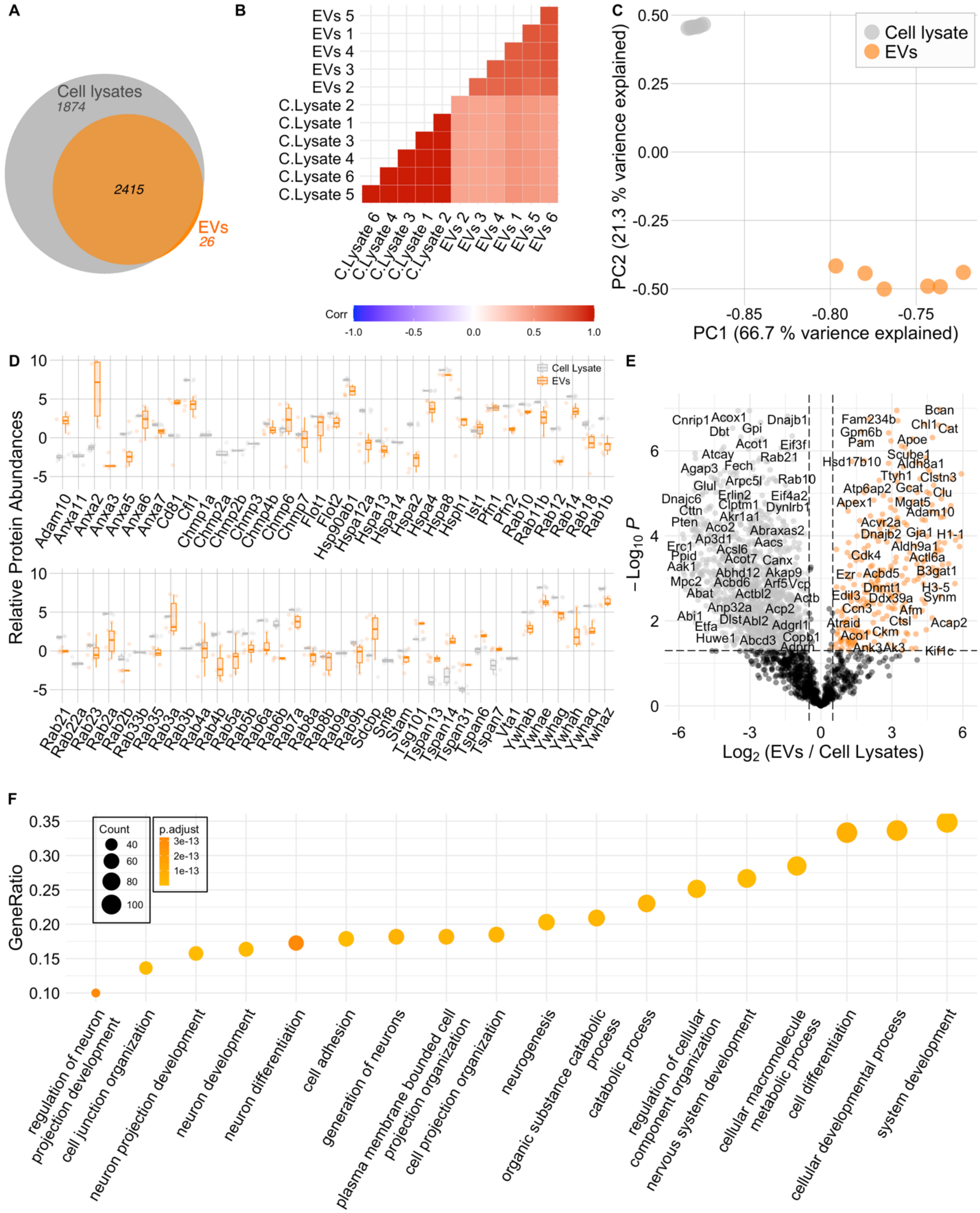
A
Cell lysates
1874
2415
EVs
26
B
EVs 5
EVs 1
EVs 4
EVs 3
EVs 2
C.Lysate 2
C.Lysate 1
C.Lysate 3
C.Lysate 4
C.Lysate 6
C.Lysate 5
C.Lysate 6 C.Lysate 4 C.Lysate 3 C.Lysate 1 C.Lysate 2 EVs 2 EVs 3 EVs 4 EVs 1 EVs 5 EVs 6
Corr
-1.0 -0.5 0.0 0.5 1.0
C
Cell lysate
EVs
PC2 (21.3 % varience explained)
PC1 (66.7 % varience explained)
0.50 0.25 0.00 -0.25 -0.50
-0.85 -0.80 -0.75
D
Cell Lysate
EVs
Relative Protein Abundances
Adam10 Anxa11 Anxa2 Anxa3 Anxa5 Anxa6 Anxa7 Cd81 Cfl1 Chmp1a Chmp2a Chmp2b Chmp3 Chmp4b Chmp6 Chmp7 Flot1 Flot2 Hsp90ab1 Hspa12a Hspa13 Hspa14 Hspa2 Hspa4 Hspa8 Hsph1 Ist1 Pfn1 Pfn2 Rab10 Rab11b Rab12 Rab14 Rab18 Rab1b
Rab21 Rab22a Rab23 Rab2a Rab2b Rab33b Rab35 Rab3a Rab3b Rab4a Rab4b Rab5a Rab5b Rab6a Rab6b Rab7a Rab8a Rab8b Rab9a Rab9b Sdcbp Snf8 Stam Tsg101 Tspan13 Tspan14 Tspan31 Tspan6 Tspan7 Vta1 Ywhab Ywhae Ywhag Ywhah Ywhaq Ywhaz
E
−Log10 P
Log2 (EVs / Cell Lysates)
Cnrip1 Acox1 Dbt Gpi Dnajb1 Acot1 Eif3f Atcay Rab21 Agap3 Fech Glul Arpc5l Rab10 Dnajc6 Erlin2 Eif4a2 Cttn Clptm1 Dynlrb1 Pten Akr1a1 Aco2 Abraxas2 Ercc1 Ap3d1 Aacs Ppid Acsl6 Aak1 Acot7 Canx Abhd12 Akap9 Mpc2 Acbd6 Arf5 Vcp Abat Actbl2 Actb Abi1 Anp32a Acp2 Etfa Dlst Abl2 Adgrl1 Huwe1 Abcd3 Copb1 Adprh
Bcan Fam234b Chl1 Cat Gpm6b Pam Apoe Scube1 Hsd17b10 Aldh8a1 Ttyh1 Clstn3 Atp6ap2 Gcat Clu Apex1 Mgat5 Adam10 Acvr2a Dnajb2 Gja1 H1-1 Aldh9a1 Cdk4 Actl6a Ezr Acbd5 B3gat1 Dnmt1 H3-5 Edil3 Ddx39a Synm Ccn3 Afm Atraid Ctsl Acap2 Aco1 Ckm Ank3 Ak3 Kif1c
F
GeneRatio
Count
40 60 80 100
p.adjust
3e-13 2e-13 1e-13
regulation of neuron projection development
cell junction organization
neuron projection development
neuron development
neuron differentiation
cell adhesion
generation of neurons
plasma membrane bounded cell projection organization
cell projection organization
neurogenesis
organic substance catabolic process
catabolic process
regulation of cellular component organization
nervous system development
cellular macromolecule metabolic process
cell differentiation
cellular developmental process
system development

Figure 4

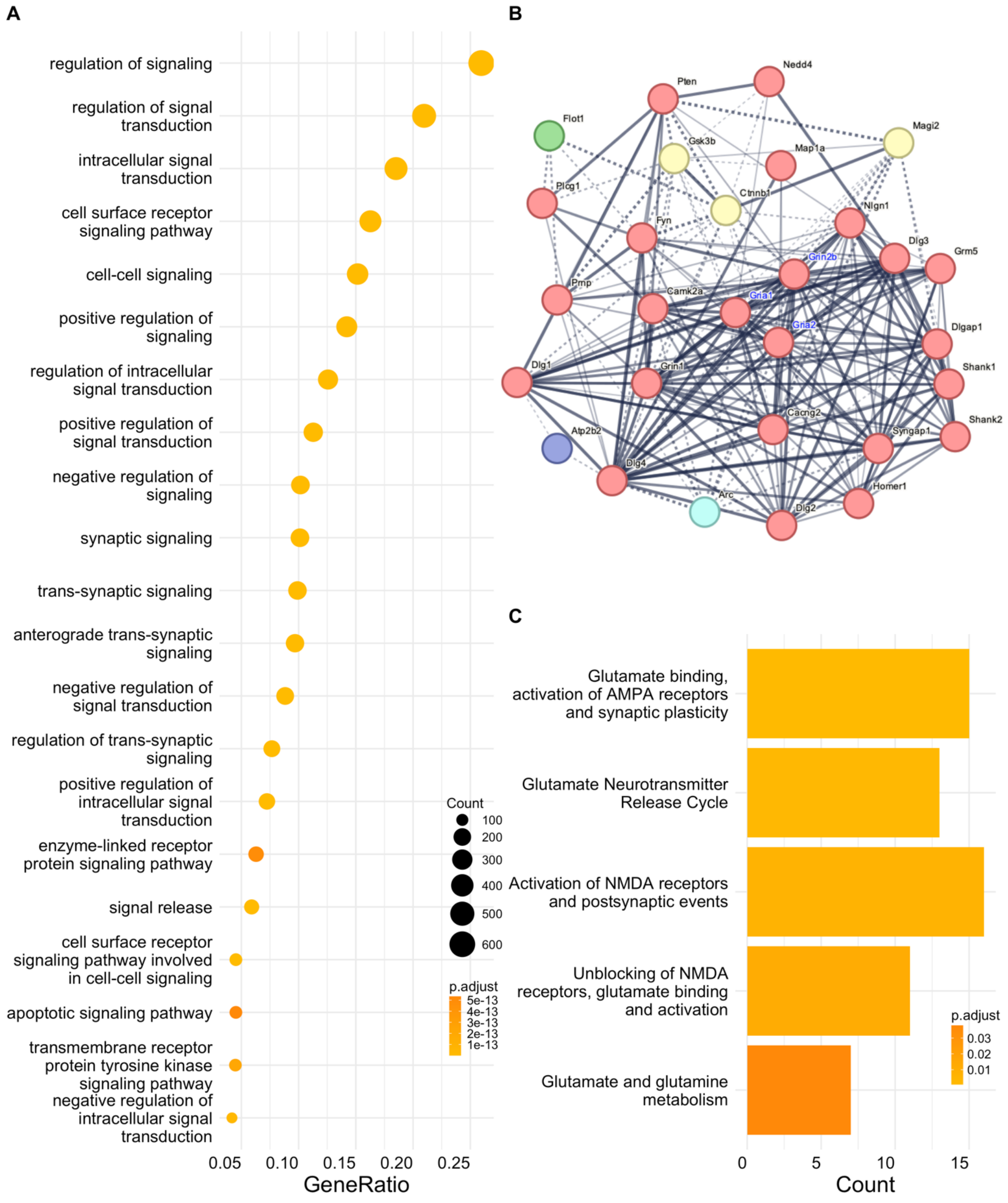
A
regulation of signaling
regulation of signal transduction
intracellular signal transduction
cell surface receptor signaling pathway
cell-cell signaling
positive regulation of signaling
regulation of intracellular signal transduction
positive regulation of signal transduction
negative regulation of signaling
synaptic signaling
trans-synaptic signaling
anterograde trans-synaptic signaling
negative regulation of signal transduction
regulation of trans-synaptic signaling
positive regulation of intracellular signal transduction
enzyme-linked receptor protein signaling pathway
signal release
cell surface receptor signaling pathway involved in cell-cell signaling
apoptotic signaling pathway
transmembrane receptor protein tyrosine kinase signaling pathway
negative regulation of intracellular signal transduction
0.05 0.10 0.15 0.20 0.25
GeneRatio
Count
100
200
300
400
500
600
p.adjust
5e-13
4e-13
3e-13
2e-13
1e-13
B
Nedd4
Pten
Flot1
Gsk3b
Map1a
Magi2
Plcg1
Ctnnb1
Nlgn1
Fyn
Dlg3
Grm5
Grin2b
Pmp
Camk2a
Gria1
Gria2
Dlgap1
Dlg1
Grin1
Shank1
Cacng2
Atp2b2
Shank2
Syngap1
Dlg4
Homer1
Arc
Dlg2
C
Glutamate binding, activation of AMPA receptors and synaptic plasticity
Glutamate Neurotransmitter Release Cycle
Activation of NMDA receptors and postsynaptic events
Unblocking of NMDA receptors, glutamate binding and activation
Glutamate and glutamine metabolism
0 5 10 15
Count
p.adjust
0.03
0.02
0.01

Figure 5

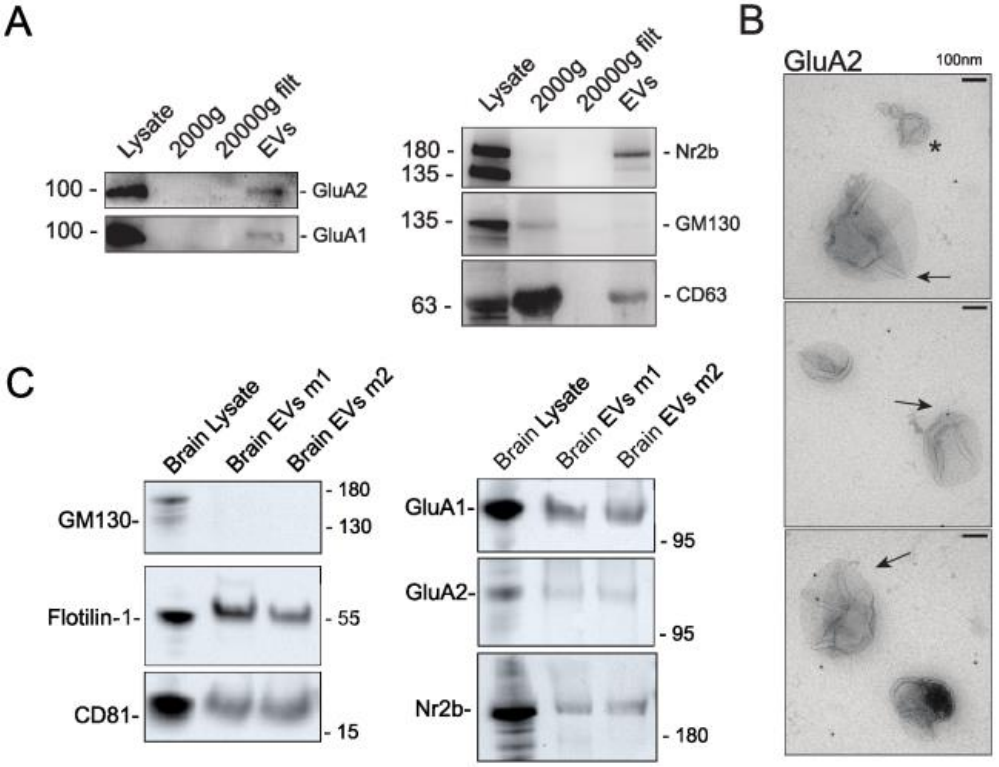
A
Lysate
2000g
20000g filt
EVs
100 -
- GluA2
100 -
- GluA1
180 -
135 -
- Nr2b
135 -
- GM130
63 -
- CD63
B
GluA2
100nm
*
C
Brain Lysate
Brain EVs m1
Brain EVs m2
GM130-
- 180
- 130
Flotilin-1-
- 55
CD81-
- 15
GluA1-
- 95
GluA2-
- 95
Nr2b-
- 180

Figure 6

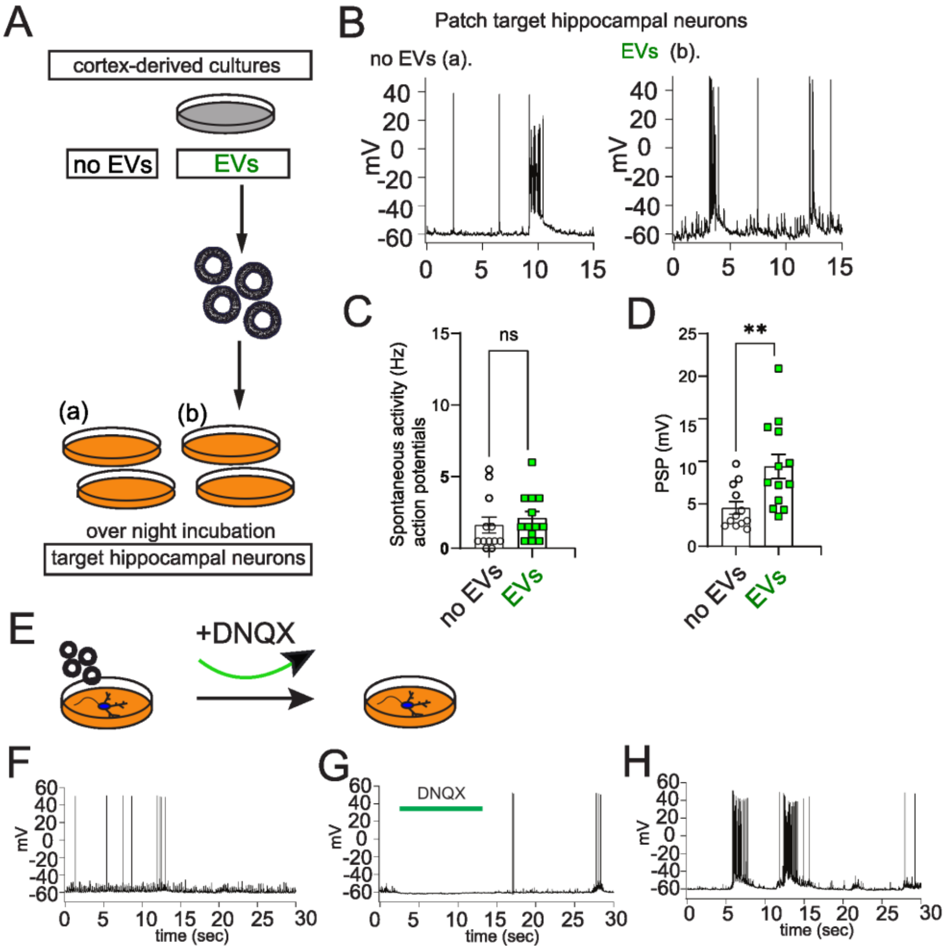
A
cortex-derived cultures
no EVs
EVs
(a)
(b)
over night incubation
target hippocampal neurons
B
Patch target hippocampal neurons
no EVs (a).
EVs (b).
mV
C
Spontaneous activity (Hz) action potentials
ns
no EVs
EVs
D
PSP (mV)
**
no EVs
EVs
E
+DNQX
F
G
DNQX
H
mV
time (sec)

Supplementary Figure S1

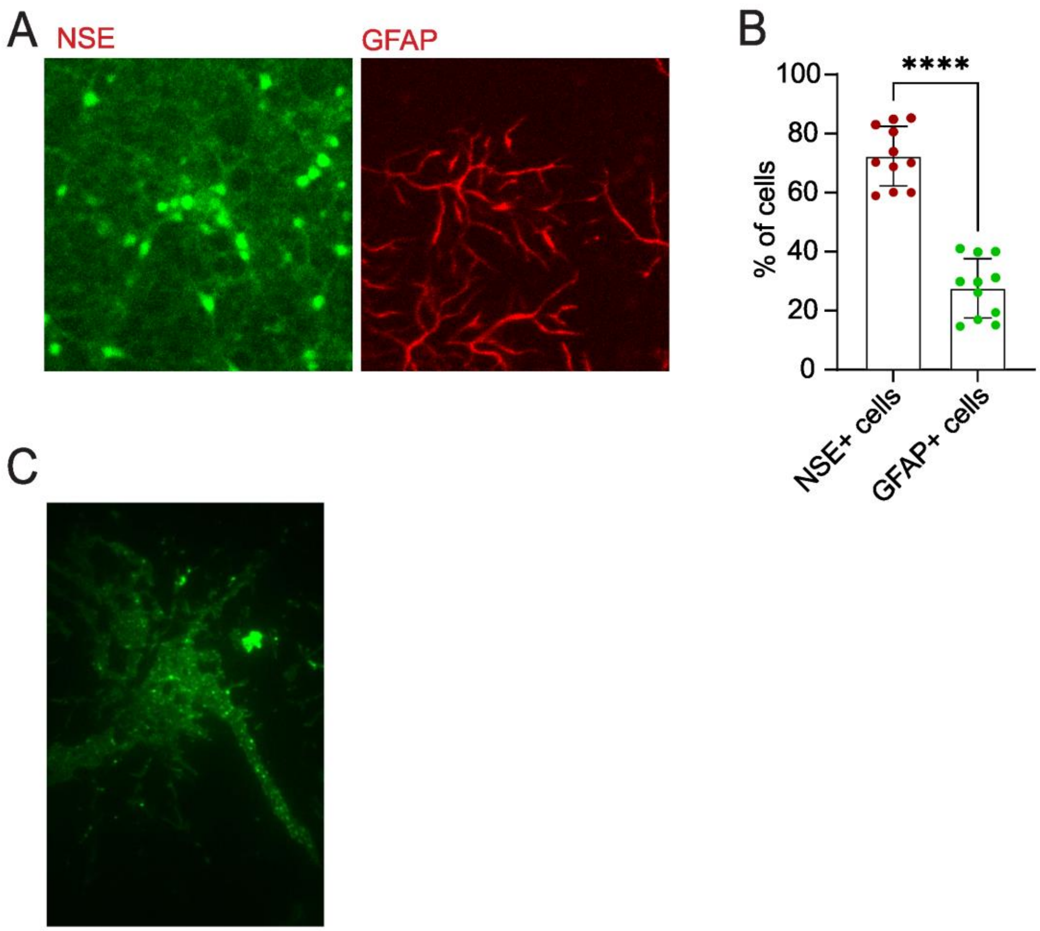

535 Supplementary Figure S2

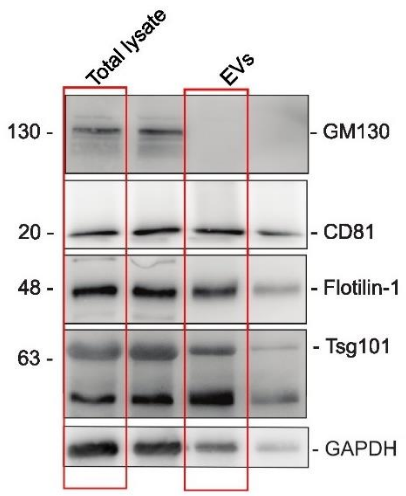

Supplementary Figure S3

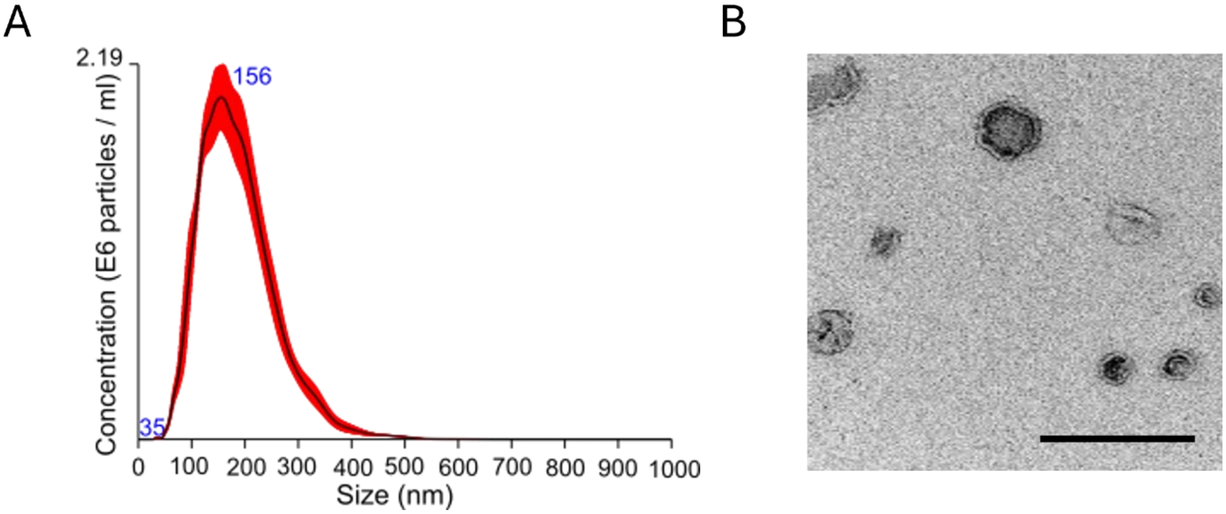

Supplementary Table S1: List of 26 EVs proteins detected uniquely in the proteome of EVs.

No.	Symbol	Description
1	Aox2	aldehyde oxidase 2
2	Fn1	fibronectin 1
3	Qsox1	quiescin Q6 sulfhydryl oxidase 1
4	Sulf2	sulfatase 2
5	Anxa3	annexin A3
6	Fam20c	FAM20C, golgi associated secretory pathway kinase
7	Uroc1	urocanase domain containing 1
8	Znf428	zinc finger protein 428
9	Pold1	polymerase (DNA directed), delta 1, catalytic subunit
10	Cemip	cell migration inducing protein, hyaluronan binding
11	Rbbp6	retinoblastoma binding protein 6, ubiquitin ligase
12	Macroh2a2	macroH2A.2 histone
13	Cdk1	cyclin-dependent kinase 1
14	Wscd1	WSC domain containing 1
15	Galnt16	polypeptide N-acetylgalactosaminyltransferase 16
16	Chga	chromogranin A
17	Hacl1	2-hydroxyacyl-CoA lyase 1
18	Hs6st3	heparan sulfate 6-O-sulfotransferase 3
19	Ext1	exostosin glycosyltransferase 1
20	Fbln1	fibulin 1
21	Krt72	keratin 72
22	Ehhadh	enoyl-Coenzyme A, hydratase/3-hydroxyacyl Coenzyme A dehydrogenase
23	Scaf8	SR-related CTD-associated factor 8
24	C3	complement component 3
25	Aldh1a7	aldehyde dehydrogenase family 1, subfamily A7
26	Fv4	retrovirus-related Env polyprotein from Fv-4 locus
